# Physical and physiological effects of game based and running based High Intensity Interval Training with equal volume: a 4 week quasi experimental study

**DOI:** 10.7717/peerj.20962

**Published:** 2026-03-27

**Authors:** Hakan Karabıyık, Tugay Durmuş, Özkan Güler, Oğuz Gürkan, Süleyman Bilgin, Dicle Aras, Veli Volkan Gürses, Mitat Koz, Mehmet Gülü, Hakan Yapici, Sameer Badri AL-Mhanna, Onur Mutlu Yasar, Monira I. Aldhahi

**Affiliations:** 1 Department of Coaching Education, Faculty of Sport Sciences, Ankara University, Ankara, Turkey; 2 Department of Coaching Education, Faculty of Sport Sciences, Yozgat Bozok University, Yozgat, Turkey; 3 Department of Coaching Education, Faculty of Sport Sciences, Adıyaman University, Adıyaman, Turkey; 4 Department of Coaching Education, Faculty of Sport Sciences, Bandırma Onyedi Eylül University, Balıkesir, Turkey; 5 Department of Physiotherapy and Rehabilitation, Faculty of Health Sciences, Eastern Mediterranean University, Famagusta, North Cyprus, Cyprus; 6 Department of Sports Management, Faculty of Sport Sciences, Kırıkkale University, Kırıkkale, Turkey; 7 Department of Recreation, Faculty of Sport Sciences, Kırıkkale University, Kırıkkale, Turkey; 8 Department of Physiology, School of Medical Sciences, Universiti Sains Malaysia, Kelantan, Malaysia; 9 Faculty of Health Sciences, Izmir Democracy University, İzmir, Turkey; 10 Department of Rehabilitation Sciences, College of Health and Rehabilitation Sciences, Princess Nourah bint Abdulrahman University, Riyadh, Saudi Arabia

**Keywords:** High intensity interval training, Small sided games, Soccer, Heart rate, Football

## Abstract

**Purpose:**

High-intensity interval training (HIIT) is a well-established exercise method, known for its effectiveness in improving various aspects of fitness. This study aimed to assess the effect of five distinct HIIT protocols, including Small Sided Games Long Interval (SSG(LI)), Long Interval (LI), Small Sided Games Short Interval (SSG(SI)), Short Interval (SI), and Sprint Interval Training (SIT), on aerobic, agility, vertical jump performance, sprinting abilities, and heart rate variability (HRV) in amateur soccer players.

**Methods:**

Forty participants were randomly assigned to one of five training groups, and a four-week training intervention was conducted. The pre- and post-training assessments included measurements of maximal oxygen uptake (VO_2max_), anaerobic power, reactive agility, vertical jump height, sprint times, and HRV parameters.

**Results:**

Results showed that all training groups demonstrated significant improvements in VO_2max_. Reactive agility performance significantly improved in the SSG(LI), SSG(SI), SI, and SIT groups, whereas no significant changes were observed in sprint times or anaerobic power. Leg-specific vertical jump performance showed significant improvements in the right and left legs. HRV parameters showed variable responses to the training protocols. Significant main effects of Time were observed for LF (*F* = 3.592, *p* = 0.016, *ηp*^2^ = 0.310) and low frequency/high frequency (LF/HF) ratio (*F* = 5.699, *p* = 0.023, *ηp*^2^ = 0.151), indicating overall changes across participants from pre- to post-training.

**Conclusions:**

These findings underscore the adaptability of HIIT and its association with improvements in aerobic endurance, agility, and leg-specific vertical jumping capabilities across different training forms, while acknowledging that causation cannot be definitively established due to the quasi-experimental design.

## Introduction

Interval training has a well-established history, with some sources indicating that this method has been utilized since the early 1900s (Foster et al., 2025; Oniz & Gocer, 2024). Evidence suggests that interval training has been employed as a structured training technique for over a century. High intensity interval training (HIIT) is a type of exercise that involves alternating short bursts of intense activity with brief periods of rest or low intensity exercise (Billat, 2001). HIIT has gained substantial attention in recent years as a time-efficient alternative to traditional endurance-based training models. Although HIIT sessions are generally shorter in duration, numerous studies have demonstrated that they can elicit physiological adaptations comparable to, or in some cases greater than, those achieved through longer bouts of conventional training (Chang et al., 2022; Poon et al., 2024). Unlike moderate-intensity continuous training (MICT), which is performed at a steady and submaximal effort, HIIT is characterized by repeated intervals of high-intensity activity that typically reach ≥80% of maximal heart rate (HR_max_) or maximal oxygen uptake (VO_2max_), interspersed with short recovery periods. This intermittent structure creates substantial cardiovascular and metabolic demand, thereby promoting improvements in aerobic capacity, cardiometabolic health, and exercise performance within a relatively shorter time frame (Buchheit & Laursen, 2013).

Numerous studies have examined the use of HIIT as a primary exercise method and investigated its effects on aerobic and anaerobic capacity (Akgül, 2019; Arazi et al., 2017; Karayiğit et al., 2020), performance markers in both individual and team sports (Delextrat, Gruet & Bieuzen, 2018; Monks et al., 2017; Stankovic et al., 2023; Karabiyik et al., 2021), health benefits (Ito, 2019; Leahy et al., 2020) and body composition (Depiazzi et al., 2019; Sultana et al., 2019; Yavuz & Dağdelen , 2021). The effectiveness of HIIT can vary depending on factors such as intensity and duration, with the literature categorizing HIIT into several methods. Two of the main methods—repeated sprint training (RST) and sprint interval training (SIT)—are based on supramaximal intensity. RST consists of repeating sprints between 3–10 s with passive recoveries of 60 s or less. In cases where the rest period is over 60 s, it should be noted that the method is intermittent sprinting and not RST (Bishop, Girard & Mendez-Villanueva, 2011). SIT typically consists of maximal or near-maximal sprint efforts lasting 20–30 s, followed by recovery periods ranging from 1 to 4 min. Although SIT is classified as a subtype of HIIT, the literature frequently compares SIT with traditional HIIT protocols. Within this framework, comparative studies report that both modalities substantially improve cardiorespiratory fitness, while SIT demonstrates greater efficacy in reducing body weight and body mass index (BMI) (Naves et al., 2018). Furthermore, both HIIT and SIT elicit similar cardiovascular and metabolic adaptations to endurance training (Wood et al., 2016). SIT in the present study should not be interpreted as a separate or competing method from HIIT, but rather as a specific subtype within the broader HIIT framework. To avoid misinterpretation, the intention of these references is to highlight that different HIIT configurations—including supramaximal sprint-based protocols such as SIT—can elicit similar physiological adaptations despite differences in format, rather than suggesting that SIT stands outside the HIIT classification.

Clarifying the definition of ‘supramaximal’ training is essential. Although some authors argue that exertion beyond 100% of an individual’s maximal capacity is physiologically unattainable, brief efforts performed at velocities exceeding those associated with VO_2max_ are indeed possible, albeit unsustainable. Such intensities surpass the anaerobic threshold and depend predominantly on anaerobic metabolic pathways, providing the physiological rationale for categorizing them as supramaximal. This concept is relevant when considering the intensity of short-interval (SI) and long-interval (LI) training protocols. SI training involves repeating high-intensity efforts (90–105% of maximal aerobic speed) for 10-60 s, with equally brief recovery periods. One study suggested that SI training can replicate the physiological effects of slower, continuous running, while incorporating high-intensity intervals (Wallner et al., 2014). In contrast, long-interval training consists of longer intervals (2–5 min) at 80–95% of maximum aerobic speed (MAS) with either passive or active recovery periods (Rønnestad et al., 2020).

“Game-based HIIT” has emerged as an alternative training approach that integrates interval training principles with sport-specific scenarios (Laursen & Buchheit, 2019). Small-sided games (SSGs) have proven to be an efficient training method, addressing various fitness needs while also enhancing skill development, decision-making, and tactical awareness (Carlsson et al., 2025). SSGs have been shown to improve cardiovascular fitness, agility, and sport-specific endurance, while allowing players to refine technical and tactical skills in a game-like environment (De Souza et al., 2024). Studies have indicated that SSGs lead to similar improvements in physical performance, alongside enhanced sport-specific movements and technical skills, such as basketball shooting (Zeng et al., 2022). As a result, SSGs are increasingly utilized by both amateur and professional teams for conditioning purposes (Clemente, Martins & Mendes, 2014; Sgrò et al., 2018). The benefits of SSGs or training involving the ball are often assessed based on achieving an exercise intensity of approximately 90–95% of HR_max_. This approach is believed to enhance sport-specific endurance, target muscles used in the game, refine technical and tactical skills under game-specific conditions, and ultimately facilitate an effective transition to actual match performance (Chamari et al., 2005; Hoff et al., 2002). The intensity of SSGs drills can be adjusted or customized to elicit various physical, technical, and tactical outcomes through several factors. These factors include the number of players participating (Da Silva et al., 2011), dimensions and layout of the playing area (Kelly & Drust, 2009), duration of exercise and rest intervals (Sampson, Fullagar & Gabbett, 2015), specific game rules (Hill-Haas et al., 2010), coaching instructions and motivation (Sahli et al., 2020), and the scoring system employed (Pulling, Twitchen & Pettefer, 2016). Manipulating these variables allows coaches to modify SSGs according to their training objectives and desired player development outcomes.

Despite extensive research on HIIT, limited studies have systematically compared different interval methods, particularly in SSGs and their effects on fitness, skill development, and sport-specific performance. There is a need to examine how SI and LI protocols, as well as all-out sprint interval training, affect fitness components in running- and game-based formats. This study therefore aimed to compare five distinct HIIT protocols to clarify their relative effectiveness, providing practical guidance for optimizing conditioning programs in both individual and team sports contexts.

Based on the existing literature and the characteristics of different HIIT methods, the following hypotheses were established for the present study:

(i) All HIIT protocols would improve aerobic performance, agility, and vertical jump ability.

(ii) Short-interval and long-interval protocols, when applied in both running- and game-based forms, would yield similar performance adaptations.

(iii) The supramaximal SIT protocol would elicit greater improvements in anaerobic and heart rate variability (HRV) parameters compared with the planned percentage-based interval protocols.

## Materials & Methods

### Participants

The required sample size was calculated using G*Power software (version 3.1.9.4; Heinrich Heine University, Düsseldorf, Germany). The calculation was based on detecting a medium effect (*f* = 0.20) on VO_2max_, with a significance level of 0.05 and a statistical power of 0.80. Effect size estimates were derived from previous studies examining the impact of HIIT on cardiorespiratory fitness Based on these parameters, the minimum required sample size was determined to be 40 participants. Therefore, the study included 40 amateur soccer players (excluding goalkeepers) with a mean age of 23  years (±2.8  years). The inclusion criteria for the study were defined as having a medical report confirming good health, no serious lower extremity injuries in the past 12 months, and not using any regular medication or supplements that could affect performance. All participants were actively competing male amateur soccer players who were recruited from university-level soccer teams and local amateur clubs. They had been training regularly for at least three sessions per week during the competitive season, with a minimum of 3 years of playing experience in organized soccer. Participants were screened to ensure they maintained consistent physical activity levels and were free of any performance-affecting conditions. Participants were randomly assigned to one of five groups using a computer-generated randomization method, with baseline VO_2max_ values used to stratify participants and ensure balanced cardiorespiratory fitness across groups. The groups included: SSGLI, LI, SSG(SI), SI, and SIT. To control for potential confounding factors, participants were required to refrain from using any regular medications or supplements that could affect their performance during the study. The study protocol received approval from the Internal Review Board for Research with Human Subjects at Kırıkkale University (2023.02.03) and adhered to the ethical guidelines outlined in the Code of Ethics of the World Medical Association Declaration of Helsinki (Association, 2013). All participants provided written informed consent prior to enrolment in the study.

All training and testing procedures were performed under standardized and calibrated conditions to ensure reliability and reproducibility.

### Study design

The study employed a parallel five-group, randomized, and repeated measures design to compare the effects of five alternative HIIT protocols (SSG(LI), LI, SSG(SI), SI, SIT) with a similar training volume. The interventions were conducted at the end of the soccer season to ensure that there were no other training effects on players. The study was completed over a total of 6 weeks, consisting of pre-tests, post-tests, and a four-week training period. Pre- and post-tests included measurements of HRV, body composition, the 30-15 Intermittent Fitness Test (30-15 IFT), running-based anaerobic sprint test (RAST), reactive agility test (RAT), 30-meter sprint test, and vertical jump tests. The participants underwent a series of tests on three different days. On the first test day, they completed HRV measurements, followed by body composition assessments, and concluded with the 30-15 IFT. RAST was completed on the second test day. The third test day involved a sequence of tests, starting with the RAT, followed by the 30-meter sprint test and concluding with jumping tests. A 5-minute recovery period was provided between tests on the final test day. Training sessions began at least 24 h after the pre-tests, and post-tests were conducted with a 48-hour break after the final training session. The training sessions were conducted over four weeks, three days a week, with a minimum of 24 h between sessions.

### Tests

All pre- and post-tests were conducted in a controlled environment sports facility (20.6–21.6 °C, 33–42% relative humidity). Since all participants were familiar with the procedures due to their affiliation with their respective teams, they were already familiar with all protocols. Participants were informed not to consume caffeine or alcohol within 12 h before the tests, to maintain their regular dietary programs, to avoid over-the-counter medications or supplements within 24 h, and not to engage in strenuous activities within 48 h before the tests. To minimize the impact of diurnal variations, both pre- and post-tests were conducted at the same time of the day (±1 h) and in the same order in the same sports facility for each participant.

### Heart rate variability (HRV)

For the measurements, individuals were asked to lie supine on a classic stretcher with their upper bodies exposed. Participants were instructed to remove their shoes, socks, and any metal objects on their person. Next, four clip electrodes (limb electrodes) were applied with gel to their metal parts, and cable input ends were positioned above the wrists and ankles. Three suction electrodes (Wilson electrodes) were placed in the chest. The first electrode was positioned on the right side of on the chest, in the transverse plane over the 5th rib and sagittal plane right in the middle of the sternum and the chest nipple; the second electrode was placed on the left side of the chest, in the transverse plane between the 4th and 5th ribs and to the lateral edge of the sternum; the third electrode was positioned on the left side of the chest, in the transverse plane over the 5th rib, and at the anterior border of the midaxillary line. The participants were instructed not to speak or move during the measurement, and the data were recorded for 5 min under these conditions. After the measurement, the time and frequency domain parameters related to HRV, including low-frequency power (LF), high-frequency power (HF), normalized high-frequency power (HFnu), the LF/HF ratio, and the root mean square of successive differences (RMSSD).

### Anthropometrics

Anthropometric and body composition analyses included height, body weight, and body fat percentage measurements. Height was measured using a stadiometer (Seca Stadiometer 213, Seca, Birmingham, UK) with a precision of one mm. Body weight was measured using a bioimpedance device (AVIS 333 PLUS; Jawon Medical, Korea).

Anthropometric measurements were performed by two trained researchers following standardized protocols. All measurements were completed on the same day for each participant to minimize variability. Interrater reliability was assessed by having both researchers independently measure a subset of participants, yielding intraclass correlation coefficients (ICCs) above 0.95 for all variables. Field-based methods were applied carefully, with repeated measurements taken when necessary, to ensure high validity and reliability of the data.

Skinfold measurements (Holtain, Crosswell, UK) were performed to determine body fat percentage. These measurements were taken following the guidelines of Jackson and Pollock (Jackson & Pollock, 1985), focusing on the chest, abdomen, and thigh in men and on the right side of the body. Body density values for men were calculated using the SUM3 skinfold equation developed by Jackson and Pollock (Jackson & Pollock, 1978). Subsequently, body density values were converted to %BF (percentage of body fat) using Siri’s formula (Siri, 1956): %BF = [(4.95/body density) ×4.50] ×100.

### 30-15 Intermittent fitness test (30-15 IFT)

The 30-15 IFT involves a series of 30-second shuttle runs followed by 15 s of passive recovery. The initial running speed is set at eight km/h for the first 30 s run and then increases by 0.5 km/h for each subsequent 45 s stage. Participants shuttle back and forth between two lines placed 40 m apart, guided by a prerecorded beep. This pacing mechanism helps participants time their runs appropriately and adjust their speed as they approach 3-meter zones at each end of the course and in the middle (at the 20-meter line), signaled by a short beep. During the 15 s recovery interval, each player walked forward to the nearest of the three lines, either in the middle or at one end of the running area, depending on where the previous stage was completed. This places them in the upcoming stage. The test was concluded when a player was unable to maintain the prescribed running speed or failed to reach a 3-meter zone around each line at the moment of the audio signal on three consecutive occasions. In cases where participants could not complete a stage, their score was recorded as the last successfully completed stage, and their running speed at that stage was documented as their maximal 30-15 IFT running velocity (RV) (Buchheit, 2008; Buchheit, 2010). The following formula is used to estimate VO_2max_ based on the final running speed, where RV is the final running speed, G stands for sex (female = 2; male = 1), A is age, and W is weight (Buchheit, 2010). VO_2max_ (ml kg^−1^ min−1) = 28.3−(2.15 × G)−(0.741 × A) −(0.0357 × W) + (0.0586 × A × RV) + (1.03 × RV). Anaerobic Sprint Reserve (ASR) was calculated as the difference between the maximal sprinting velocity and maximal aerobic speed (Buchheit, 2010). In this context, the maximal sprinting velocity was calculated as the speed (km h^−1^) obtained from the 30-meter sprint test. At the end of the test, participants’ perceived exertion levels were recorded according to the Borg CR10 scale (Borg, 1998).

### Running based anaerobic sprint test (RAST)

The RAST was used to evaluate the anaerobic power (Nobari et al., 2023). Sprint times were recorded using wireless single-beam timing gates (Smart Speed; Fusion Sport, Queensland, Australia). Each participant executed six consecutive 35-meter sprints at their maximum speed, with a 10 s rest period between each repetition. After completing the test, the following parameters were calculated: Power = Weight × Distance^2^ ÷ Time^3^, Peak Power = the highest power measurement, Average Power = the sum of all six power values ÷ 6, fatigue index (FI) = (Best sprint−Worst sprint) ÷ Best sprint (Draper & Whyte, 1997).

### Reactive agility test

In our study, we employed the Y-shaped agility test known as the RAT. This assessment is both valid (Oliver & Meyers, 2009; Lockie et al., 2014a) and reliable (Oliver & Meyers, 2009) for evaluating planned changes in direction and reactive agility performance. The testing procedures for changes in single-beam agility have been well established in the literature (Lockie et al., 2014a; Lockie et al., 2014b). The RAT was conducted using wireless, single beam timing gates (SmartSpeed, Fusion Sport, Queensland, Australia) positioned 1.0 meter above the ground, with precision recording to the nearest 0.01 s. The timing gates were arranged in a 1-1-2 formation. To ensure accuracy, a goniometer was used to measure the 45° angle from the center of the trigger gate to the middle of the target gate. The participants initiated the test from a stationary position positioned 30 cm behind the initial timing gate. They were instructed to sprint maximally toward the first set of gates and visually identify the right or left gate emitting a flash, indicating the next gate to sprint through. Four maximal RAT trials were randomly performed, with a 30 s break enforced between each sprint. During the break, the participants walked back to the starting line and rested passively before commencing the next sprint. The best time among the four RAT trials was selected for subsequent analyses.

### Sprint test

To evaluate sprint performance, we conducted tests indoors, simultaneously measuring times at 5, 10, and 30 m. Each participant completed two trials, with a 3-minute interval of slow walking and rest between each sprinting effort. Participants were instructed to initiate sprints from a stationary starting position, placing their preferred foot 0.5 m behind the starting line. Accurate timing for the 5-, 10-, and 30-meter sprints was achieved using infrared photoelectric cells (Smart Speed, Fusion Sport, Queensland, Australia), with a precision of 0.001 s. The best results obtained from these trials were selected for subsequent analysis.

### Vertical jump tests

The participants began by performing jumps with their right, left, and both legs, while keeping their hands unrestricted. Subsequently, they underwent a countermovement jump (CMJ) test with their hands placed on their hips. For the CMJ test, participants were asked to complete three maximal jumps on a jump mat (SmartJump; Fusion Sport, Sumner Park, QLD, Australia). Following each set of three maximal jumps, a 3-minute rest interval was provided. In the hand-on-hip countermovement jumps, the participants executed a controlled movement with the hip region, avoiding any arm movement, to reach an upright position. A countermovement jump involves a swift downward motion, followed by an explosive vertical leap, aiming for the maximum height. The same jumping technique was applied to the free-arm CMJs, except that participants were not required to keep their hands on their hips (Gülü & Akalan, 2021). Jump height (cm) was recorded during the jump test. To assess the right-left difference, the value obtained from the right leg was subtracted from that obtained from the left leg. A positive difference indicates that the right leg jumped higher than the left leg, whereas a negative difference suggests that the left leg performed better than the right leg.

### Intervention protocols

All the training sessions were conducted exclusively in a synthetic football field. Training sessions were carried out for a total of 4 weeks, occurring three days a week, totaling 12 sessions, with a minimum of 24 h of rest between sessions. Specific training times were scheduled for each group, and the sessions were consistently held at the same time of the day (±1 h). Throughout the training sessions, all athletes were instructed to wear the Catapult Sports™ Optimeye X4 GPS transmitters (South Melbourne, Victoria, Australia). These 10-Hz GPS devices (Catapult Sports Optim Eye X4) were strategically placed between the scapulae within a specially designed undergarment worn beneath the athletes’ playing jerseys. To ensure minimal inter-unit error between devices, each athlete used the same GPS unit for the entire duration of the training period (Nobari et al., 2023; Draper & Whyte, 1997). Real-time Catapult tracking was used primarily in SSG conditions to ensure athletes achieved the required intensity despite the dynamic and unpredictable nature of gameplay. In the running groups (LI and SI), intensity was controlled through predetermined individual target distances based on MAS, which allowed consistent pacing without requiring live feedback from the GPS system. The GPS devices were still worn in these groups to verify post-session compliance, while verbal cues were provided continuously to ensure athletes met the prescribed intensity. During the training sessions, perceived exertion levels were assessed at the end of each repetition using the modified Borg Scale ranging from 0 to 10 (Oliver & Meyers, 2009). Except for the SIT group, in the other four groups, the intensity of the training sessions increased by 5% in the last two weeks compared to the first two weeks of training. The SIT group was encouraged to perform each repetition with an all-out effort. A standard warm-up lasting for 5 min was conducted at the beginning of each training session, followed by a 5-minute cool down at the end. For the running groups, training intensity was calculated using the formula: Intensity = MAS (m/s) × training duration (s) × training intensity (Lockie et al., 2014a). The SSG(LI) group conducted four *vs.* four small-sided games in a 21 × 21 m area (increased to 22 × 22 m in the last two weeks) with no goalkeepers. These games consisted of two minutes of exertion followed by two minutes of rest, and they were performed for three repetitions. The LIs group engaged in 100-meter sprints in a dedicated area. In the first two weeks, they performed these sprints at approximately 90% effort, which increased to approximately 95% effort in the last two weeks. The sprints were executed with 2 min of exertion followed by 2 min of rest, repeated for three sets. The SSG(SI) group played 2 *vs.* 2 small-sided games in a 15 × 15 m area (expanded to 16 × 16 m in the last two weeks). These games involved 1 min of exertion followed by 1 min of rest, and they were repeated for six sets. The SI group conducted a 100-meter repeat at approximately 100% effort in the first two weeks, which was increased to approximately 105% effort in the last two weeks. These repeats were performed with 1 min of exertion followed by 1 min of rest, repeated for six sets. The SIT group performed 15 s all out sprints with 2 min of rest between each sprint, repeated for eight sets (Table 1). To control training intensities, running groups (LI, SI) monitored running distances within the specified time frames, whereas game groups used real-time tracking from the GPS system and heart rate monitoring. Supervisors overseeing the sessions provided cues to ensure that prescribed intensity levels were maintained. When considering the entire training process from warm-up to cool-down, the protocol volumes were as follows: SSG(LI) 20 min, LI 20 min, SSG(SI) 21 min, SI 21 min, and SIT 20 min (Table 2).

**Table 1:** Characteristics of HIIT methods.

**HIIT**	**Duration**	**Intensity**	**Rest**	**Recovery**
Repeated Sprint Training (RST)	6–10 s	All-Out	>60 s	Passive
Sprint Interval Training (SIT)	10–30 s	All-Out	1–4 min	Passive
Short interval (SI)	10–60 s	90–105% of MAS	10-60s	Short=Passive Long= 45% (of MAS)
Long interval (LI)	2–5 min	80–95% of MAS	1–4 min	Short=Passive Long= 45% (of MAS)
Small Sided Games (SSGs)	2–5 min	Adjustable	1:30–2 min	Passive

**Notes.**

s, second; min, minute; MAS, maximal aerobic speed.

**Table 2:** Training characteristics for each group.

**Group**	**First 2 Weeks**	**Last 2 Weeks**
**SSG (LI)**	21*21 m(4*4) ((∼55 m^2^) 2 min*2 min (3 reps) (∼90%) WU : 5 min, CD : 5min (20 min)	21*21 m(4*4) ((∼55 m^2^) 2 min*2 min (3 reps) (∼95%) WU : 5 min, CD : 5 min (20 min)
**LI**	2 min*2 min (3r eps) (∼90%) WU : 5 min, CD : 5 min (20min)	2 min*2 min (3 reps) (∼95%) WU : 5 min, CD : 5 min (20min)
**SSG (SI)**	15*15 m (2*2) ((∼56 m^2^) 1 min*1 min (6 reps) (∼100%) WU : 5min, CD : 5 min (21 min)	16*1 6m (2*2) (∼64 m^2^) 1 min*1 min (6 reps) (∼105%) WU : 5min, CD : 5 min (21 min)
**SI**	1min*1 min (6 reps) (∼100%) WU : 5 min, CD : 5 min (21min)	1min* 1min (6 reps) (∼105%) WU : 5 min, CD : 5 min (21min)
**SIT**	15 s*2 min (8 reps) (all-out) WU : 5 min, CD : 5 min (20 min)	15 s*2 min (8 reps) (all-out) WU : 5 min, CD : 5 min (20 min)

**Notes.**

SSG(LI) Small Sided Games Long Interval LI Long Interval SSG (SI) Small Sided Games Short Interval SI Short Interval SIT Sprint Interval Training WU Warm up CD Cool Down

The training volumes were nearly equalized in this manner. During the training period, data such as the distance covered by athletes (m; m/min), max speed (km/h), HR_avg_ (beats/min), HR_max_ (beats/min), HR_exertion_ (a.u.), and Player Load (a.u.) were recorded using the GPS system software. HR_exertion_ is a heart-rate derived metric that represents the cumulative cardiovascular load relative to the duration and intensity of the activity. Player Load is a Catapult-based external load measure calculated from tri-axial accelerometry, representing the summed mechanical stress from accelerations, decelerations, and changes of direction. Additionally, the rate of perceived exertion (RPE) was obtained using the Borg Scale. These values were averaged for each training session to determine the mean training values (Table 2).

### Statistical analysis

The experimental data for the total sample are expressed as means and standard deviations (mean ± SD). Statistical analysis was initially performed using the Shapiro–Wilk normality test. Mauchly sphericity test was used to test the homogeneity of variances and Greenhouse–Geisser correction was applied when necessary. A two-way mixed model analysis of variance (ANOVA) for repeated measures was used to test for main and interaction effects of group (levels: SSG(LI), LI, SSG(SI), SI, SIT) and timing of measurement (levels: pre-intervention, post-intervention) for each outcome variable independently. In line with standard analytical governance, follow-up comparisons were executed using Bonferroni-adjusted procedures when significant main or interaction effects were identified. Effect sizes were quantified *via* partial eta squared (*η*^2^*p*), and interpreted according to established quantitative benchmarks (0.01–0.06 = small, 0.06–0.14 = medium, and 0.14 ≤ large) (Cohen, 2013). The level of significance was set at *p* < 0.05. All statistical calculations were performed using IBM^®^ SPSS^®^ version 22 statistical package for Windows and Excel 2021 (Microsoft Corp., Redmond, WA, USA).

## Results

Table 3 shows performance metrics for each training group during the first two weeks (first 6 sessions) and the last two weeks (last 6 sessions) of the intervention, allowing evaluation of workload and physiological responses across the training period. SSG(LI), LI, SSG(SI), and SIT groups showed an increase in the total steady state (“steady state” refers to the endurance-related capacity of the athletes to maintain a continuous submaximal exercise intensity over time without a progressive increase in physiological stress. In other words, it reflects the players’ ability to sustain a stable internal load (*e.g.*, heart rate and perceived exertion) during prolonged activity, which is indicative of improved aerobic efficiency). Most groups increased their average speed, while the SI group maintained a similar speed level across training weeks. SSG(LI) and SSG(SI) groups showed an increase in average heart rate. LI, SI, and SIT groups showed decreases in maximum heart rate. A substantial decrease in HR_exertion_ was observed in the SSG(LI) group across the training period, while the SIT group consistently showed markedly lower HR_exertion_ values compared to the other training modalities. All groups experienced an increase in RPE, except for the SIT group.

Table 4 presents an analysis of the performance parameters before and after the training protocols.

**Table 3:** Descriptive analysis of each training protocol.

**Parameters**	**Firs/Last**	**SSG (LI)**	**LI**	**SSG (SI)**	**SI**	**SIT**
**Total distance (m)**	**First 6**	784.69 ± 51.43	1,460.87 ± 115.8	817.49 ± 33.95	1,634.7 ± 92.92	733 ± 35.99
	**Last 6**	804.03 ± 63.8	1,512.37 ± 121.55	857.05 ± 53.97	1,624.48 ± 90.15	762.11 ± 26.55
**Total Distance (m/min)**	**First 6**	130.78 ± 8.57	243.48 ± 19.3	136.25 ± 5.66	272.45 ± 15.49	122.17 ± 6
	**Last 6**	134 ± 10.63	252.06 ± 20.26	142.84 ± 9	270.75 ± 15.03	127.02 ± 4.43
**Max Speed (km/h)**	**First 6**	16.92 ± 1.51	19.29 ± 1.58	16.26 ± 0.65	21.18 ± 0.69	26.85 ± 1.34
	**Last 6**	16.89 ± 1.08	19.2 ± 1.21	16.42 ± 0.62	20.78 ± 0.54	26.94 ± 1.41
**HRavg (beat/min)**	**First 6**	154.11 ± 21.59	165.05 ± 8.85	148.91 ± 31.51	172.51 ± 7.18	155.96 ± 9.76
	**Last 6**	159.38 ± 16.51	161.77 ± 9.76	161.94 ± 8.02	169.46 ± 6.92	155.04 ± 11.68
**HRmax (beat/min)**	**First 6**	169.37 ± 21.92	180.46 ± 8.75	160.73 ± 33.81	185.92 ± 6.51	175.75 ± 12.35
	**Last 6**	176.02 ± 15.53	178.12 ± 9.75	177.9 ± 8.24	183.53 ± 5.65	173.28 ± 12.93
**HRexertion (a.u.)**	**First 6**	907.56 ± 281.71	762.25 ± 220.56	770.52 ± 206.14	910.42 ± 206.53	189.53 ± 37.24
	**Last 6**	526.94 ± 169.38	684.52 ± 178.53	612.39 ± 179.66	808.66 ± 133.03	170.8 ± 51.34
**Player Load (a.u.)**	**First 6**	100.44 ± 11.24	151.45 ± 21.5	110.89 ± 26.76	159.87 ± 19.29	82.59 ± 14.12
	**Last 6**	99.29 ± 12.14	154.74 ± 20.74	121.68 ± 7.57	156.74 ± 23.16	82.62 ± 11.7
**RPE**	**First 6**	5.48 ± 0.62	7.49 ± 0.58	4.99 ± 1.31	7.27 ± 0.85	5.94 ± 1.85
	**Last 6**	6.28 ± 1.13	7.62 ± 0.54	5.3 ± 0.51	7.31 ± 0.82	5.8 ± 0.75

**Notes.**

SSG (LI) Small Sided Games Long Interval LI Long Interval SSG (SI) Small Sided Games Short Interval SI Short Interval SIT Sprint Interval Training

**Table 4:** Analysis of performance parameters for each training group.

**Parameters**	**Pre Post**	**SSG (LI)**	**LI**	**SSG (SI)**	**SI**	**SIT**	**Time**	**Group**	Group ^*^ Time
**Age (years)**	** **	23.27 ± 2.29	23.31 ± 2.33	22.58 ± 2.91	23.27 ± 4.09	21.48 ± 1.8			
**Body composition**									
**Height (cm)**		176.13 ± 4.09	175.29 ± 7.43	172.57 ± 4.76	177 ± 7.15	178.14 ± 8.61			
**Body Weight (kg)**	**Pre**	73.24 ± 9.59	78.43 ± 16.22	70.61 ± 6.95	73.95 ± 7.23	78.29 ± 12.95	*F* = 0.159 *p* = 0.692 *η*p2 = 0.005	*F* = 0.713 *p* = 0.589 *η*p2 = 0.082	*F* = 1.69 *p* = 0.177 *η*p2 = 0.174
	**Post**	73.79 ± 9.18	77.79 ± 15.75	70.56 ± 7.19	73.39 ± 6.46	79.54 ± 13.93			
**Body Fat (%)**	**Pre**	15.86 ± 2.79	20.9 ± 13.44	16.57 ± 3.91	16.69 ± 2.86	16.63 ± 4.29	*F* = 2.568 *p* = 0.119 *η*p2 = 0.074	*F* = 0.459 *p* = 0.765 *η*p2 = 0.054	*F* = 1.401 *p* = 0.256 *η*p2 = 0.149
	**Post**	16.31 ± 2.55	17.6 ± 7.1	16.64 ± 3.28	16.01 ± 2.88	15.7 ± 3.51			
**Heart rate variability**									
**HF**	**Pre**	168.63 ± 93.68	836 ± 635.34	675.29 ± 877.35	998.13 ± 864.73	981.43 ± 1,099.46	*F* = 0.494 *p* = 0.487 *η*p2 = 0.015	*F* = 2.479 *p* = 0.064 *η*p2 = 0.237	*F* = 0.730 *p* = 0.578 *η*p2 = 0.084
	**Post**	451.25 ± 367.2	1,011.86 ± 1,244.56	312 ± 200.6	1,353.38 ± 991.24	1,048.29 ± 639.88			
**Hfnu**	**Pre**	29.64 ± 10.57	40 ± 17.35	25.89 ± 13.22	51.9 ± 20.61	44.6 ± 28.16	*F* = 3.430 *p* = 0.073 *η*p2 = 0.097	*F* = 2.091 *p* = 0.105 *η*p2 = 0.207	*F* = 2.309 *p* = 0.079 *η*p2 = 0.224
	**Post**	40.94 ± 17.83	51.09 ± 20.56	33.31 ± 12.68	39.8 ± 14.21	55.14 ± 21.18			
**LF**	**Pre**	421.38 ± 303.16	1,181 ± 890.51	1,316 ± 1,308.42	810.63 ± 703.35	913.71 ± 661.46	*F* = 0.259 *p* = 0.614 *η*p2 = 0.008	*F* = 2.224 *p* = 0.088 *η*p2 = 0.218	*F* = 3.592 *p* = 0.016 *η*p2 = 0.310
	**Post**	611 ± 432.4	598.43 ± 270.27	654.71 ± 343.1	1,690.88 ± 853.44^*^	702.14 ± 305.54			
**LF/HF**	**Pre**	2.76 ± 1.31	1.96 ± 1.39	4.47 ± 4.31	1.22 ± 0.91	3.58 ± 4.92	*F* = 5.699 *p* = 0.023 *η*p2 = 0.151	*F* = 1.200 *p* = 0.330 *η*p2 = 0.130	*F* = 1.602 *p* = 0.198 *η*p2 = 0.167
	**Post**	1.87 ± 1.15	1.4 ± 1.46	2.66 ± 2.2	1.87 ± 1.18	1.11 ± 0.92^*^			
**RMSSD**	**Pre**	39.13 ± 19.13	70.29 ± 25.38	58.43 ± 30.69	72.25 ± 27.78	56.29 ± 28.05	*F* = 2.729 *p* = 0.108 *η*p2 = 0.079	*F* = 1.493 *p* = 0.227 *η*p2 = 0.157	*F* = 1.613 *p* = 0.195 *η*p2 = 0.168
	**Post**	62.13 ± 34.69	75.86 ± 48.1	45.57 ± 9.41	80.75 ± 33.35	70.57 ± 30.08			
**30-15 Intermittent fitness test**									
**MAS (km/h)**	**Pre**	18.5 ± 1.93	17.57 ± 1.48	19.21 ± 1.41	17.75 ± 1.25	17.57 ± 1.3	*F* = 53.985 *p* = 0.000 *η*p2 = 0.628	*F* = 1.104 *p* = 0.372 *η*p2 = 0.121	*F* = 1.316 *p* = 0.285 *η*p2 = 0.141
	**Post**	19.63 ± 2.33^*^	18.93 ± 2.03^*^	20.07 ± 1.67^*^	19.81 ± 1.07 *	18.79 ± 1.25^*^			
**ASR (km/h)**	**Pre**	6.15 ± 1.92	7.73 ± 1.18	5.48 ± 0.97	6.88 ± 1.46	6.81 ± 1.55	*F* = 53.122 *p* = 0.000 *η*p2 = 0.624	*F* = 1.537 *p* = 0,0.215 *η*p2 = 0.161	*F* = 2.633 *p* = 0.056 *η*p2 = 0.248
	**Post**	4.84 ± 2.04^*^	5.92 ± 2.00^*^	4.81 ± 1.03	4.21 ± 1.40^*^	5.6 ± 1.65^*^			
**MSS (km/h)**	**Pre**	24.65 ± 1.22	25.3 ± 0.82	24.69 ± 0.82	24.63 ± 1.15	24.39 ± 1.28	*F* = 3.377 *p* = 0.075 *η*p2 = 0.095	*F* = 0.668 *p* = 0.619 *η*p2 = 0.077	*F* = 1.583 *p* = 0.203 *η*p2 = 0.165
	**Post**	24.47 ± 0.54	24.84 ± 1.09	24.88 ± 0.99	24.02 ± 1.02	24.38 ± 1.48			
**VO**_**2max**_ ** (ml/kg/min)**	**Pre**	50.66 ± 5.21	48.14 ± 3.73	52.08 ± 3.27	48.75 ± 3.27	47.58 ± 2.86	*F* = 51.913 *p* = 0.000 *η*p2 = 0.619	*F* = 1.295 *p* = 0.293 *η*p2 = 0.139	*F* = 1.395 *p* = 0.258 *η*p2 = 0.148
	**Post**	53.31 ± 6.20^*^	51.35 ± 4.75^*^	54.08 ± 3.92^*^	53.7 ± 3.17^*^	50.34 ± 2.93^*^			
**RPE**	**Pre**	8 ± 1.2	8.71 ± 0.76	8 ± 0.82	7.38 ± 2.39	7.57 ± 0.98	*F* = 39.354 *p* = 0.000 *η*p2 = 0.552	*F* = 0.396 *p* = 0.810 *η*p2 = 0.047	*F* = 2.518 *p* = 0.061 *η*p2 = 0.239
	**Post**	9.38 ± 1.19^*^	9.29 ± 0.76	8.71 ± 1.5	9.75 ± 0.46^*^	9.71 ± 0.49^*^			
**Running anaerobic sprint test**									
**Average Power (W)**	**Pre**	556.23 ± 123.7	575.97 ± 88.37	563.75 ± 94.4	535.39 ± 78.97	535.02 ± 95.71	*F* = 0.041 *p* = 0.841 *η*p2 = 0.001	*F* = 0.277 *p* = 0.890 *η*p2 = 0.034	*F* = 0.273 *p* = 0.893 *η*p2 = 0.033
	**Post**	534.42 ± 90	577.69 ± 82.06	554.21 ± 76.98	541.99 ± 73.34	546.29 ± 65.8			
**Peak Power (W)**	**Pre**	685.94 ± 156.95	744.64 ± 110.44	690.85 ± 92.6	694.31 ± 104.17	674.73 ± 145.58	*F* = 0.190 *p* = 0.666 *η*p2 = 0.006	*F* = 0.213 *p* = 0.929 *η*p2 = 0.026	*F* = 0.439 *p* = 0.779 *η*p2 = 0.052
	**Post**	669.7 ± 109.13	709.87 ± 117.33	677.44 ± 99.79	693.34 ± 140.74	705.31 ± 118.63			
**Fatigue Index (%)**	**Pre**	7.19 ± 2.69	8.94 ± 2.37	7.65 ± 1.51	8.68 ± 2.6	7.23 ± 2.56	*F* = 1.836 *p* = 0.185 *η*p2 = 0.054	*F* = 0.447 *p* = 0.774 *η*p2 = 0.053	*F* = 0.595 *p* = 0.669 *η*p2 = 0.069
	**Post**	7.1 ± 1.94	7.49 ± 2.52	6.76 ± 1.9	7.81 ± 3.91	7.66 ± 2.66			
**Reactive agility test**									
**Reactive Agility (s)**	**Pre**	1.61 ± 0.13	1.42 ± 0.08	1.59 ± 0.07	1.47 ± 0.12	1.62 ± 0.15	*F* = 27.106 *p* = 0.000 *η*p2 = 0.459	*F* = 3.575 *p* = 0.016 *η*p2 = 0.309	*F* = 1.403 *p* = 0.255 *η*p2 = 0.149
	**Post**	1.43 ± 0.19^*^	1.4 ± 0.17	1.46 ± 0.11^*^	1.29 ± 0.12^*^	1.44 ± 0.12^*^			
**Sprint test**									
**5 m (ms)**	**Pre**	1.08 ± 0.06	1.05 ± 0.09	1.1 ± 0.06	1.11 ± 0.05	1.11 ± 0.09	*F* = 0.053 *p* = 0.819 *η*p2 = 0.002	*F* = 1.331 *p* = 0.280 *η*p2 = 0.143	*F* = 0.863 *p* = 0.496 *η*p2 = 0.097
	**Post**	1.06 ± 0.05	1.02 ± 0.1	1.08 ± 0.09	1.11 ± 0.08	1.21 ± 0.36			
**10 m (ms)**	**Pre**	1.84 ± 0.09	1.78 ± 0.1	1.85 ± 0.07	1.85 ± 0.08	1.83 ± 0.09	*F* = 3.233 *p* = 0.082 *η*p2 = 0.092	*F* = 1.033 *p* = 0.405 *η*p2 = 0.114	*F* = 1.430 *p* = 0.247 *η*p2 = 0.152
	**Post**	1.85 ± 0.05	1.8 ± 0.12	1.84 ± 0.09	1.89 ± 0.1	1.99 ± 0.35			
**30 m (ms)**	**Pre**	4.39 ± 0.22	4.27 ± 0.14	4.38 ± 0.15	4.39 ± 0.21	4.44 ± 0.23	*F* = 3.321 *p* = 0.078 *η*p2 = 0.094	*F* = 0.692 *p* = 0.603 *η*p2 = 0.080	*F* = 1.526 *p* = 0.218 *η*p2 = 0.160
	**Post**	4.42 ± 0.1	4.35 ± 0.2	4.35 ± 0.18	4.5 ± 0.19	4.44 ± 0.27			
**Vertical jump test**									
**Bilateral HoW (cm)**	**Pre**	35.42 ± 3.58	38 ± 5.78	36.58 ± 6.21	36.76 ± 4.07	35.96 ± 4.39	*F* = 1.188 *p* = 0.284 *η*p2 = 0.036	*F* = 0.758 *p* = 0.560 *η*p2 = 0.087	*F* = 0.900 *p* = 0.476 *η*p2 = 0.101
	**Post**	34.47 ± 4.24	39.9 ± 4.07	37.77 ± 9.08	38.03 ± 4.02	35.66 ± 3.91			
**Bilateral HF (cm)**	**Pre**	41.72 ± 3.34	45.09 ± 6.86	42.1 ± 5.48	45.73 ± 6.56	41.83 ± 6.78	*F* = 2.159 *p* = 0.151 *η*p2 = 0.063	*F* = 0.983 *p* = 0.431 *η*p2 = 0.109	*F* = 1.456 *p* = 0.238 *η*p2 = 0.154
	**Post**	39.33 ± 3.36	45.41 ± 5.26	42.55 ± 8.35	43.63 ± 4.25	41.75 ± 5.85			
**Left (cm)**	**Pre**	22.03 ± 2.52	23.59 ± 4.17	20.16 ± 3.42	22.89 ± 2.34	20.64 ± 3.48	*F* = 10.492 *p* = 0.003 *η*p2 = 0.247	*F* = 1.557 *p* = 0.210 *η*p2 = 0.163	*F* = 0.463 *p* = 0.762 *η*p2 = 0.055
	**Post**	22.74 ± 3.5	25.42 ± 4.2	22.5 ± 3.36^*^	24.32 ± 4.1	21.5 ± 2.63			
**Right (cm)**	**Pre**	20.29 ± 2.34	23.65 ± 5.46	21.81 ± 4.46	22.9 ± 2.32	21.83 ± 5.05	*F* = 4.780 *p* = 0.036 *η*p2 = 0.130	*F* = 1.246 *p* = 0.311 *η*p2 = 0.135	*F* = 0.395 *p* = 0.610 *η*p2 = 0.047
	**Post**	22.11 ± 2.27	25.41 ± 2.94	23.44 ± 1.71	23.61 ± 2.62	21.9 ± 4.54			
**Right-Left Diff. (cm)**	**Pre**	−9.35 ± 13.4	−1.3 ± 12.3	6.27 ± 15	−1.14 ± 16.4	3.25 ± 17.45	*F* = 0.001 *p* = 0.974 *η*p2 = 0.000	*F* = 0.835 *p* = 0.513 *η*p2 = 0.095	*F* = 0.550 *p* = 0.701 *η*p2 = 0.064
	**Post**	−2.8 ± 11.05	0.23 ± 8.09	3.86 ± 13.8	−3.96 ± 21.13	−0.04 ± 14.77			

**Notes.**

SSG (LI) Small Sided Games Long Interval LI Long Interval SSG (SI) Small Sided Games Short Interval SI Short Interval SIT Sprint Interval Training HoW Hand on Waist HsF Hands Free

* *p* < 0,05.

**Figure 1:**
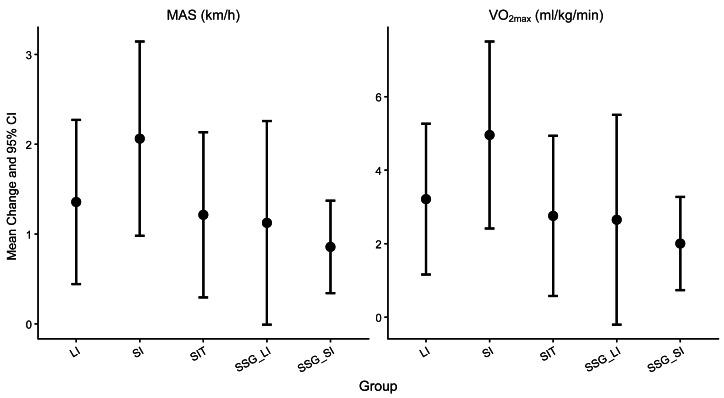
Within-group mean changes and 95% CIs in MAS and VO_2max_ following four weeks of running based and game based high-intensity interval training.

Changes in maximal aerobic speed and VO_2max_ varied across training groups, with improvements observed across all interventions and the magnitude of change differing between running based and game based HIIT formats (Fig. 1).

There was no significant effect on body composition, running anaerobic sprint test, or sprint test results.

**Figure 2:**
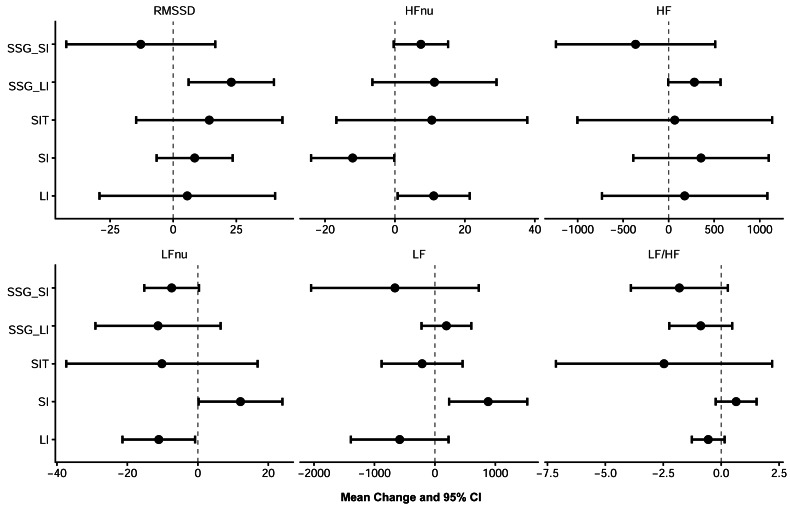
Within-group mean changes and 95% CIs in time and frequency domain HRV indices following four weeks of running based and game based high-intensity interval training.

Changes in LF and LF/HF ratios varied across training groups, with the direction and magnitude of change suggesting increases in LF mainly in the SI group and decreases in the LF/HF ratio primarily in the SIT group (Fig. 2).

## Discussion

Overall, the results of our study strongly support all the three hypotheses. First, there were no significant differences in the effects of planned short and long intervals with a specific percentage of intensity when compared with an all-out sprint interval protocol. This implies that individuals can select their preferred interval method without expecting significant variations in the outcomes. Second, we found no significant difference between short- and long-interval methods when comparing the game-based and running-based forms of exercise. This suggests that individuals and coaches can choose either short or long intervals based on their preferences and training goals, without anticipating substantial differences in outcomes. Third, we observed no significant differences among the various HIIT methods; however, within each method, significant intragroup improvements were evident based on defined characteristics. This indicates that, while HIIT methods may not differ significantly in overall effectiveness, individuals can still expect substantial improvements tailored to their specific training goals within their chosen method.

While it is often associated with improved anaerobic fitness and cardiovascular health, HIIT can also improve aerobic endurance. Our study demonstrated significant improvements in aerobic endurance in all groups. All training methods showed a significant increase in VO_2max_ from pre-to post training. One study compared the effects of two different HIIT protocols with steady state training on aerobic and anaerobic capacity and found similar effects with our study. The results showed significant improvements in VO_2max_ and peak power output for all three training groups, with no significant differences between them (Foster et al., 2015). Another study investigated the effects of 4-week low volume SIT on aerobic and anaerobic performance in canoe polo athletes. The study findings suggest that the SIT group had a significant increase in VO_2peak_, anaerobic threshold, peak, and mean power output (Sheykhlouvand et al., 2018).

Similar to the referenced study, VO_2_max significantly increased in the present investigation. However, unlike their findings, we did not observe improvements in anaerobic threshold or peak and mean power output following the intervention. The primary focus of this study was to compare various HIIT methods and reveal potential differences in their effects. However, it is worth noting that several studies have investigated the effects of altering the load and rest ratios within the same training protocols. The effects of two different work-to-rest ratio configurations of the HIIT were compared in one study. Both groups showed significant improvements in all anaerobic and aerobic performance measures, with no significant differences between the two groups. These findings suggest that both 10:5s-HIIT and 20:10s-HIIT can induce similar performance adaptations (Moghaddam et al., 2021). Our study findings also suggest parallel results, as all groups showed significant improvements in VO_2max_ despite the difference in loading times of the training protocols used in the study. We also found greater RPE levels in all training groups in post 30-15 IFT. SSG-LI, SI, and SIT groups showed significant improvements in RPE after the training protocols. Several studies have compared the effects of different forms of HIIT on the RPE. One study investigated acute responses to SIT and HIIT in active adults. These results were similar to our findings, as the authors concluded that SIT is more challenging and produces higher levels of RPE (Wood et al., 2016). In contrast, although RPE increased from pre- to post-training during the 30-15 IFT in the present study, the SIT group consistently reported lower exertion scores throughout the intervention compared with the LI and SI groups. This indicates that the perceptual load experienced during training may not directly reflect the performance-related improvements observed after the intervention, and direct comparison with studies examining acute responses should therefore be interpreted with caution.

Agility is defined as the ability to execute rapid whole-body movements that involve changes in both direction and speed. It is widely recognized as a crucial attribute in team sports, enabling players to evade opponents during offensive maneuvers or to use pressure on opponents when defending (Inglis & Bird, 2016; Young & Willey, 2010). The results of the RAT demonstrated significant improvements in reactive agility performance from pre-test to pos*t*-test for the SSG (LI), SSG (SI), SI, and SIT groups. It can be assumed that the improvements in reactive agility in SSGs groups may be attributed to the training provided through the game format itself. Several study outcomes encourage our findings, as one study suggests that evasive drills or small sided games simulate the unpredictability of actual game scenarios and help athletes improve their agility skills (Young & Farrow, 2013). Studies have also pointed out that SSGs designed specifically for agility training may generate superior results in matching relevant variables than pre-planned changes in sprint direction (Chaouachi et al., 2014).

Vertical jumping ability is a fundamental skill in various sports, including volleyball, basketball, and football, and enhancing an athlete’s vertical jumping capability is a significant factor contributing to their overall performance (Rodacki, Fowler & Bennett, 2002; Waller, Gersick & Holman, 2013). Although studies have reported that HIIT and SSGs improve jumping performance (Karahan, 2020; Howard & Stavrianeas, 2017; Jurišić et al., 2021), there were no statistically significant changes in the bilateral height of the jump, bilateral horizontal flight distance, or right-left difference in jump height in our study. However, our findings suggest that our training protocols had a more significant impact on leg-specific jump performance than on overall bilateral performance. The increase in right and left leg jumping performance, as well as the decrease in the right/left difference, may indicate that the protocol we applied had a corrective effect in terms of vertical jump performance.

Whether it serves as a direct factor in sports, such as track and field, or an indirect factor in team sports, the capacity to generate maximal, brief, and short-term effort, such as sprinting, is a critical parameter in numerous competitive sports (Spencer et al., 2005). In the running anaerobic sprint test, we examined several key parameters, including the Average Power (W), Peak Power (W), and Fatigue Index (%) before and after the test. There was no significant effect found on Average Power, Peak Power, or Fatigue Index in any group. Additionally, the results of the Sprint Test also indicated that there were no significant changes in sprint times at 5 m, 10 m, or 30 m distances, unlike previous studies that found greater improvements in sprint performance (Delextrat, Gruet & Bieuzen, 2018; Sperlich et al., 2011). Although there were improvements in the 5 m, 10 m, and 30 m sprint times, these differences were not statistically significant. However, our results are consistent with previous findings in the literature. It was found that neither HIIT nor SSGs had any significant effects on sprint performance (Zhang et al., 2023; Fernandez-Fernandez et al., 2012; Iacono et al., 2016).

The results of our study suggest that SI and SIT have a notable impact on various HRV parameters. LF increased significantly after SI, and the LF/HF ratio decreased significantly after SIT. There was an overall increase in parasympathetic activity, as evidenced by the HF, normalized high-frequency (Hfnu), and Root Mean Square of Successive Differences (RMSSD) values after the intervention. These improvements are interpreted as associations with the applied HIIT interventions rather than definitive causal effects due to the study design. One study had findings similar to ours, suggesting that HRV-guided HIIT training had a significant impact on parasympathetic activity (Da Silva et al., 2019). In our study, there was an increase in several HRV parameters, although this increase was not statistically significant. We believe that this increase is equivalent to an increase in the overall aerobic endurance performance.

## Conclusions

The findings demonstrate the adaptability of HIIT in improving aerobic endurance, agility, and leg-specific vertical jump performance across different training methods. Significant enhancements were observed in reactive agility for the SSG(LI), SSG(SI), SI, and SIT groups, while single-leg jump performance improved despite no change in bilateral CMJ height. These results suggest that various HIIT protocols can yield comparable outcomes, allowing coaches and athletes to select approaches based on preferences and specific training goals. Incorporating SSGs into training programs appears particularly effective, as game-like scenarios can enhance sport-specific performance components. HIIT, including SSG-based approaches, offers time-efficient conditioning that targets multiple performance attributes, making it a valuable tool for in-season training. Future research should examine the long-term effects of these protocols throughout a competitive season, their applicability to other sports and populations, and potential cognitive and psychological benefits, such as decision-making and tactical skills. A limitation of the current study is the relatively small sample size (*n* = 8 per group), which may reduce statistical power; however, participant homogeneity was maintained to ensure internal validity and comparability.

## Supplemental Information

10.7717/peerj.20962/supp-1 Supplemental Information 1 Dataset of the study

## Additional Information and Declarations

### Data Availability

The following information was supplied regarding data availability: The raw data are available in the Supplemental File.
